# Exploring the role of health consciousness and environmental awareness in purchase intentions for green-packaged organic foods: an extended TPB model

**DOI:** 10.3389/fnut.2025.1528016

**Published:** 2025-02-21

**Authors:** Youyou Li, Baoshun Shan

**Affiliations:** ^1^Department of Business Administration, Dongshin University, Naju, Republic of Korea; ^2^Department of Education, Dongshin University, Naju, Republic of Korea

**Keywords:** health consciousness, environmental awareness, green packaging, organic foods, theory of planned behavior, TPB

## Abstract

**Background:**

With the increasing rise of green consumption concepts, consumers are becoming more concerned with the health and environmental attributes of products. However, research on purchase intentions for green-packaged organic foods remains relatively limited, particularly regarding the specific impact of health consciousness and environmental awareness. There is a clear gap in the literature addressing these factors.

**Method:**

Based on the Theory of Planned Behavior (TPB), this study constructs a relationship model between health consciousness, environmental awareness, attitude, subjective norms, perceived behavioral control, and purchase intentions for green-packaged organic foods. A total of 372 valid survey samples were collected, and data analysis was performed using SPSS 26.0 and AMOS 28.0 software to validate the theoretical model and research hypotheses.

**Results:**

Structural equation modeling (SEM) analysis reveals that all hypotheses are significantly supported, and the model demonstrates good fit. Mediation effect analysis further reveals the impact of health consciousness and environmental awareness on purchase intentions, indicating that they influence consumer intentions indirectly through attitudes, subjective norms, and perceived behavioral control.

**Conclusion:**

Health consciousness and environmental awareness not only significantly affect consumers’ purchase intentions for green-packaged organic foods through direct paths but also have important indirect effects through attitudes, subjective norms, and perceived behavioral control. This study provides a new perspective for the theoretical development of green consumer behavior and offers valuable practical insights for businesses and policymakers in promoting green consumption and sustainable products.

## 1 Introduction

As global environmental issues intensify, such as climate change, resource depletion, and pollution, there is increasing societal concern for sustainable development. This trend has led consumers to gradually shift from traditional consumption patterns to more eco-friendly and sustainable consumption practices. Green consumption not only concerns environmental protection but also has profound implications for individual health. As a consumption mode centered on health and environmental protection, green consumption emphasizes that products should reflect eco-friendly and health-conscious attributes during their production, processing, and packaging stages, thus contributing to ecological protection and safeguarding consumer health (1).

In this context, green packaging and organic foods have become key topics in green consumption. Green packaging requires products to meet sustainable development standards in their packaging design and production processes, ensuring that they are environmentally friendly, harmless to human health, reusable, and recyclable (2). Organic foods, on the other hand, avoid the use of chemical pesticides, fertilizers, chemical preservatives, and synthetic substances, as well as genetic engineering techniques, ensuring that food production and processing meet environmental and health requirements (3).

The application of green packaging to organic foods, i.e., green-packaged organic foods, ensures that not only the food itself meets organic standards but also focuses on environmental protection and sustainability in the packaging process. This combination not only meets consumers’ demand for food safety and quality but also aligns with modern society’s emphasis on environmental protection and the sustainable use of resources, promoting the food industry’s transition toward greener and more sustainable practices (4). As the consumption of green packaging and organic foods continues to grow, stimulating consumers’ purchase intentions for these green products has become a key issue in green marketing and sustainable consumption promotion.

The Theory of Planned Behavior (TPB) offers an effective theoretical framework for explaining consumer behavioral intentions, demonstrating significant validity in predicting consumer purchasing behavior, especially in the context of green consumption (5, 6). According to TPB, the core psychological factors influencing purchase intentions are attitude, subjective norms, and perceived behavioral control (7). Although TPB has been widely applied in consumer behavior research, especially in the context of green consumption, the existing literature on green-packaged organic foods remains insufficient. Specifically, the roles of health consciousness and environmental awareness in such consumption behaviors have not been fully explored (8, 9).

Based on the TPB framework, this study introduces health consciousness and environmental awareness as key influencing factors, aiming to further expand the theoretical applicability and explanatory power of the TPB model. Health consciousness refers to an individual’s concern for their own health status and the tendency to take actions to maintain their health (10). As health issues become more prominent, consumers’ preferences for health-friendly products, such as green packaging and organic foods, have become increasingly significant. Environmental awareness refers to an individual’s understanding of environmental issues and their willingness to engage in environmentally protective behaviors in daily life (11). In the context of green consumption, environmental awareness not only affects consumers’ choices of eco-friendly products but also strengthens their sense of social responsibility for sustainable development (12).

Therefore, this study explores how health consciousness and environmental awareness influence consumers’ attitudes, subjective norms, and perceived behavioral control, which in turn affect their purchase intentions for green-packaged organic foods. This provides a new perspective for the theoretical development of green consumer behavior and offers practical insights for businesses’ green marketing strategies and sustainable consumption promotion.

## 2 Theoretical analysis and research hypotheses

### 2.1 Theory of planned behavior

The Theory of Planned Behavior (TPB), proposed by Ajzen in 1991, is an important theoretical framework for explaining individual behavioral intentions and actions (13). According to this theory, individual behavior intentions are jointly determined by three core factors: attitude, subjective norms, and perceived behavioral control. Attitude refers to an individual’s positive or negative evaluation of a behavior, reflecting the consumer’s subjective perception and value judgment about the behavior (14). In the context of green consumption, when consumers recognize the positive value of green purchasing behaviors, they are more likely to form positive purchase intentions (15, 16). Existing research has pointed out that consumers’ attitudes toward green packaging and organic foods significantly influence their purchasing intentions, and positive attitudes are key drivers of green buying behavior (17, 18). Subjective norms refer to the perceived social pressure, or the expectations and demands of others or societal groups regarding an individual’s behavior (19, 20). In the context of green consumption, subjective norms are manifested in the support or pressure that consumers feel from friends, family, and other social groups. Green-packaged organic foods, due to their environmental and health benefits, have broad social recognition and support (21, 22). Studies show that social initiatives and group support can effectively motivate consumers to engage in environmentally friendly purchasing behaviors, thereby enhancing their green purchase intentions (23). Several empirical studies have further validated the positive impact of subjective norms on green consumption decisions (24, 25). Thus, it is expected that subjective norms also play a significant role in purchasing decisions for green-packaged organic foods. Perceived behavioral control refers to an individual’s subjective judgment of the difficulty or ease of performing a behavior, including whether they have the necessary resources and capabilities to carry out that behavior (26). In green consumption, perceived behavioral control is influenced by factors such as product availability and convenience (27). Studies show that higher perceived behavioral control positively promotes consumers’ green purchase intentions (28). Furthermore, studies have shown that perceived behavioral control positively affects consumers’ intentions to purchase organic foods (29, 30). Therefore, when consumers feel they have the ability and conditions to purchase green-packaged organic foods, their behavioral intentions are enhanced.

Based on the above analysis, the following research hypotheses are proposed:

*H1:* Attitude positively influences consumers’ purchase intention for green-packaged organic foods.

*H2:* Subjective norms positively influence consumers’ purchase intention for green-packaged organic foods.

*H3:* Perceived behavioral control positively influences consumers’ purchase intention for green-packaged organic foods.

### 2.2 Health consciousness

With the increasing prevalence of chronic diseases and environmental pollution, consumers’ health consciousness has significantly increased, thereby influencing their consumption choices. In green consumption behavior, health consciousness has been shown to have a significant direct and indirect impact on consumers’ purchase intentions (31, 32). Organic foods, due to their avoidance of chemical pesticides and synthetic additives during production and processing, meet consumers’ demand for higher food safety and health protection (33). Moreover, green packaging, by using eco-friendly materials, further reduces food contamination risks, providing stronger health guarantees for health-conscious consumers (34). This combination of the dual advantages of green packaging and organic food’s health benefits makes it an ideal choice for consumers with high health consciousness.

Health consciousness not only directly promotes green purchase intentions but also indirectly influences consumers’ behavioral intentions by enhancing attitudes, subjective norms, and perceived behavioral control. Consumers with high health consciousness are more likely to hold positive attitudes toward organic foods because they believe these products contribute to health maintenance and long-term health benefits (35, 36). In terms of subjective norms, these consumers are more likely to be positively influenced by friends, family, and other social groups and tend to follow these external expectations in their consumption decisions (37, 38). Health consciousness also enhances consumers’ perceived behavioral control (37, 39), making them more confident and capable of purchasing green-packaged organic foods. This enhanced cognition, stemming from consumers’ understanding and recognition of the health benefits of organic foods, encourages more proactive purchasing behaviors. Green packaging not only enhances the environmental attributes of organic foods but also strengthens consumers’ positive reactions in terms of attitude, subjective norms, and perceived behavioral control (40, 41).

Based on the above analysis, the following research hypotheses are proposed:

*H4:* Health consciousness positively influences consumers’ purchase intention for green-packaged organic foods.

*H5:* Health consciousness positively influences consumers’ attitude.

*H6:* Health consciousness positively influences consumers’ subjective norms.

*H7:* Health consciousness positively influences consumers’ perceived behavioral control.

### 2.3 Environmental awareness

Environmental awareness refers to consumers’ understanding of environmental issues and their willingness to protect the environment. It plays a crucial role in green consumption. Research has shown that environmental awareness has an important role in consumer behavior, significantly promoting green purchasing behavior (12, 42). In the organic food sector, environmental awareness motivates consumers to focus more on the environmental benefits of products and whether they adhere to sustainable development principles (43). Green packaging, as a manifestation of eco-friendly concepts, uses biodegradable and recyclable materials, helping reduce environmental pollution and meeting the needs of environmentally conscious consumers (44).

In the TPB framework, environmental awareness also influences consumers’ attitude, subjective norms, and perceived behavioral control, thus modulating their behavioral intentions. Consumers with high environmental awareness typically hold more positive attitudes, seeing green products as effective tools for reducing pollution and protecting the environment, which enhances their purchase intentions (45). They are also more likely to be driven by societal expectations in subjective norms, showing support and compliance with environmentally friendly behaviors (46). Meanwhile, environmental awareness boosts consumers’ confidence and willingness to act when purchasing green-packaged organic foods, mainly due to their strong identification with the behavioral value (47). The environmental attributes of green packaging further reinforce these positive effects, making consumers’ positive reactions in terms of attitude, subjective norms, and perceived behavioral control more pronounced (40, 41).

Based on the above analysis, the following research hypotheses are proposed:

*H8:* Environmental awareness positively influences consumers’ purchase intention for green-packaged organic foods.

*H9:* Environmental awareness positively influences consumers’ attitude.

*H10:* Environmental awareness positively influences consumers’ subjective norms.

*H11:* Environmental awareness positively influences consumers’ perceived behavioral control.

### 2.4 Research model

**Figure 1:**
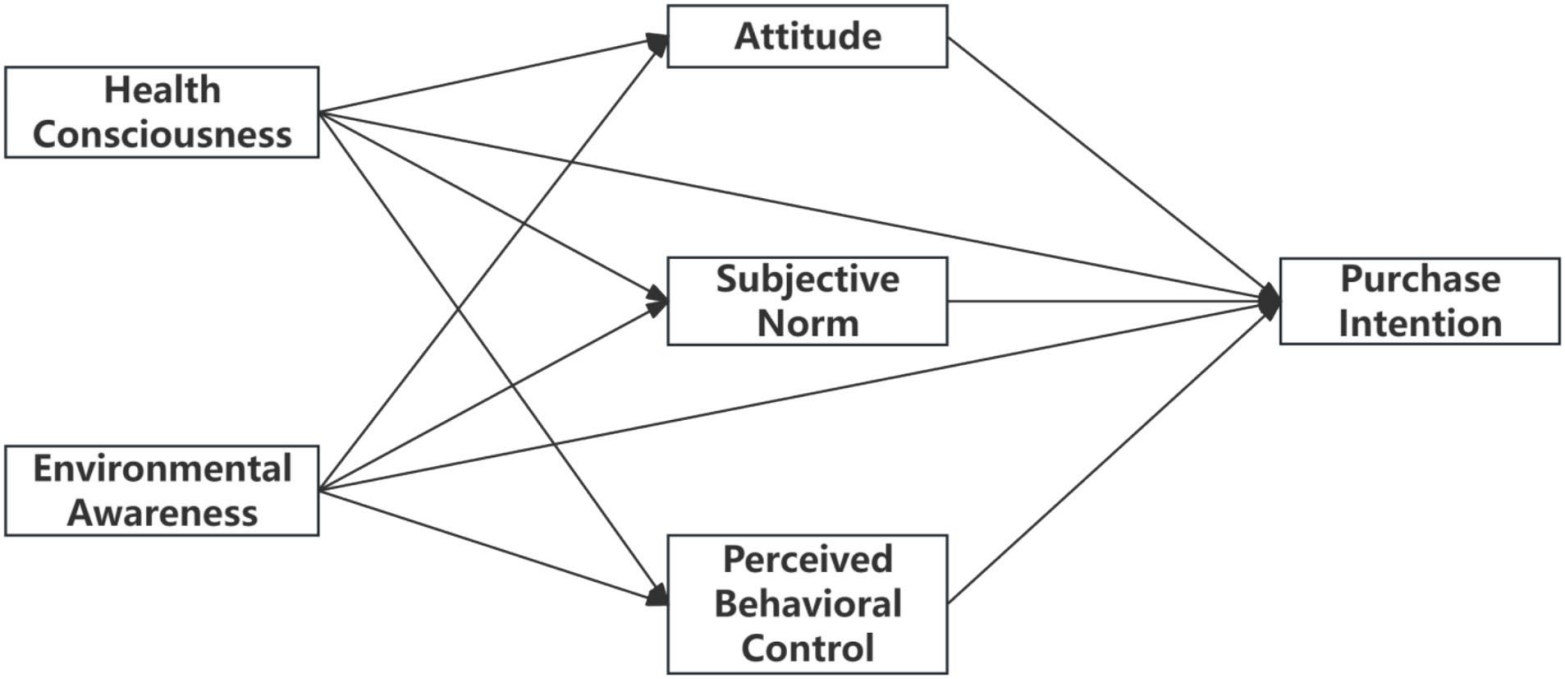
Research model.

Based on the above theoretical analysis and research hypotheses, this study constructs an expanded TPB model, as shown in Figure 1. This model introduces health consciousness and environmental awareness into the classic TPB framework to examine how these factors influence consumers’ purchase intentions for green-packaged organic foods through attitude, subjective norms, and perceived behavioral control.

## 3 Research methodology

### 3.1 Data collection

This study employed a questionnaire survey method to collect data in order to validate the research model and hypotheses. The survey questionnaires were randomly distributed through the Wenjuanxing platform (https://www.wjx.cn/) and shared on multiple popular social media platforms, reaching various regions and social groups across China. To help participants accurately understand the concept of green-packaged organic foods, relevant explanations were provided in the questionnaire to minimize potential bias in understanding. A total of 413 questionnaires were collected, with 372 valid responses after screening, resulting in an effective response rate of 90.1%.

Table 1 shows the demographic information of the respondents. The age distribution of the sample was primarily concentrated in the 26–35 years (60.22%) and 36–45 years (24.46%) age groups, representing a relatively high-consuming middle-aged demographic. The majority of respondents had a bachelor’s degree (79.03%), with 8.87% holding a master’s degree or higher, indicating a high level of education. In terms of occupation, the vast majority were company employees or managers (81.18%), and 71.77% of respondents belonged to the middle-income group with a monthly income ranging from 5,000 to 12,000 yuan.

**Table 1:** Demographic information of respondents.

Variable	Sort	Number	Percentage (%)
Gender	Male	151	40.59
	Female	221	59.41
Age	18–25	36	9.68
	26–35	224	60.22
	36–45	91	24.46
	>46	21	5.65
Educational level	High school and below	6	1.61
	Associate degree	39	10.48
	Bachelor’s degree	294	79.03
	Master’s degree and above	33	8.87
Occupation	Student/Teacher	22	5.91
	Public institution/servant	20	5.38
	Company employee/manager	302	81.18
	Freelancer/Self-employed	24	6.45
	Other	4	1.08
Monthly income	<5,000	40	10.75
	5,000–8,000	124	33.33
	8,000–12,000	143	38.44
	>12,000	65	17.47

### 3.2 Measurement items

The measurement items in this study were adapted and optimized based on existing validated scales in the literature, considering the specific context of this research. The final scale and references are presented in Table 2. Each variable includes 3 to 4 measurement items, which were rated using a 5-point Likert scale (1 = Strongly Disagree, 5 = Strongly Agree) to quantify the respondents’ level of agreement with each statement.

**Table 2:** Measurement items and references.

Variable		Measurement items	References
Health consciousness	HC1	I am very concerned about my physical health.	Yadav and Pathak (48)
	HC2	I often think about issues related to health.	
	HC3	I carefully select foods to ensure good health.	
Environmental awareness	EA1	I am very concerned about the ecological environment.	Xu et al. (46)
	EA2	I believe everyone has a responsibility to protect the environment.	
	EA3	The balance of nature is delicate and easily disrupted.	
	EA4	I am willing to control my consumption to support sustainable development.	
Attitude	A1	I think purchasing green-packaged organic food is a good idea.	Teixeira et al. (49)
	A2	I believe purchasing green-packaged organic food is a wise choice.	
	A3	I like the idea of buying green-packaged organic food.	
	A4	It would be enjoyable to buy green-packaged organic food.	
Subjective norm	SN1	My family supports purchasing green-packaged organic food.	Liang et al. (47)
	SN2	My friends support purchasing green-packaged organic food.	
	SN3	In my social circle, buying green-packaged organic food is encouraged.	
Perceived behavioral control	PBC1	I have the resources and time to purchase green-packaged organic food.	Han et al. (50)
	PBC2	If I want, I can buy green-packaged organic food.	
	PBC3	Whether to purchase green-packaged organic food is entirely up to me.	
Purchase intention	PI1	I am willing to buy green-packaged organic food when shopping.	Sun et al. (51)
	PI2	I will make an effort to purchase green-packaged organic food in the near future.	
	PI3	I intend to purchase green-packaged organic food in the near future.	
	PI4	I am willing to pay more for green-packaged organic food.	

## 4 Data analysis and results

### 4.1 Reliability and validity testing

The results of the reliability analysis show that the Cronbach’s *α* values for all latent variables are greater than 0.7, indicating good reliability of the research data. Additionally, the Kaiser-Meyer-Olkin (KMO) value is 0.915, which is above 0.8, suggesting that the data are highly suitable for factor analysis. Bartlett’s test of sphericity yielded an approximate chi-square value of 3437.474 with 210 degrees of freedom, and a *p*-value less than 0.001, further supporting the suitability of the data for factor analysis.

The results of the Confirmatory Factor Analysis (CFA) are shown in Table 3. The standardized factor loadings for health consciousness (HC), environmental awareness (EA), attitude (A), subjective norm (SN), perceived behavioral control (PBC), and purchase intention (PI) are all greater than 0.7, indicating strong correlations between the measurement items and their corresponding latent variables. The Average Variance Extracted (AVE) for each latent variable exceeds 0.5, and the Composite Reliability (CR) for each is above 0.7, demonstrating high convergent validity.

**Table 3:** Standardized loading coefficient, Cronbach’s α, AVE, and CR.

Variable		Standardized loading coefficient	Cronbach’s *α*	AVE	CR
Health consciousness	HC1	0.714	0.763	0.520	0.765
	HC2	0.722			
	HC3	0.728			
Environmental awareness	EA1	0.705	0.807	0.513	0.808
	EA2	0.724			
	EA3	0.719			
	EA4	0.719			
Attitude	A1	0.769	0.847	0.582	0.848
	A2	0.727			
	A3	0.773			
	A4	0.782			
Subjective norm	SN1	0.759	0.784	0.550	0.786
	SN2	0.734			
	SN3	0.732			
Perceived behavioral control	PBC1	0.712	0.784	0.548	0.784
	PBC2	0.774			
	PBC3	0.733			
Purchase intention	PI1	0.783	0.857	0.602	0.858
	PI2	0.751			
	PI3	0.751			
	PI4	0.816			

Table 4 presents the Pearson correlations and the square roots of the AVE for each latent variable. The results indicate that the square root of the AVE for each latent variable is greater than its correlations with other variables, demonstrating good discriminant validity between the variables.

**Table 4:** Pearson correlations and square roots of AVE.

	HC	EA	A	SN	PBC	PI
HC	0.721					
EA	0.383	0.716				
A	0.422	0.415	0.763			
SN	0.313	0.406	0.524	0.742		
PBC	0.382	0.382	0.406	0.398	0.740	
PI	0.508	0.485	0.577	0.479	0.483	0.776

### 4.2 Structural model and hypothesis testing

The results of the Structural Equation Modeling (SEM) analysis indicate that the model exhibits good fit and explanatory power. The chi-square ratio (*χ*^2^/df = 1.602) is less than 3, indicating that the model fits the data well. Other key fit indices further support the close fit between the model and the data, with an RMSEA value of 0.040, which is below the threshold of 0.10, indicating a good overall model fit. The RMR value is 0.023, which is below 0.05, suggesting small residuals and a good representation of the data structure. Furthermore, the values of GFI, AGFI, CFI, NFI, and NNFI are 0.931, 0.910, 0.968, 0.919, and 0.962, respectively, all greater than 0.90, further confirming the model’s good fit.

The results of the hypothesis testing are shown in Table 5. All path coefficients were statistically significant (*p* < 0.05), validating the effectiveness of the hypotheses. Specifically, the standardized regression coefficient for Attitude (A) on Purchase Intention (PI) was 0.294 (*p* < 0.01), indicating that attitude has a significant positive impact on purchase intention. The influence of Subjective Norm (SN) on purchase intention was 0.129 (*p* < 0.05), suggesting that social norms also play a significant role in promoting green purchase intention. The effect of Perceived Behavioral Control (PBC) on purchase intention was 0.198 (*p* < 0.01), further supporting the critical role of perceived behavioral control in the formation of behavioral intentions. Additionally, Health Consciousness (HC) had a direct effect on purchase intention with a standardized regression coefficient of 0.245 (*p* < 0.01), demonstrating a significant positive effect on green purchase behavior. Environmental Awareness (EA) influenced purchase intention with a coefficient of 0.153 (*p* < 0.05), indicating that environmental awareness also plays an important role in promoting green consumption intention. Finally, both Health Consciousness (HC) and Environmental Awareness (EA) significantly influenced Attitude (A), Subjective Norm (SN), and Perceived Behavioral Control (PBC) (*p* < 0.01), suggesting that health consciousness and environmental awareness indirectly promote the formation of purchase intention through these psychological variables.

**Table 5:** Hypothesis testing results.

Path	Standardized regression coefficient	SE	z (CR-Value)	*p*-value
A → PI	0.294	0.064	4.476	0.000
SN → PI	0.129	0.055	2.056	0.040
PBC → PI	0.198	0.077	3.081	0.002
HC → PI	0.245	0.098	3.254	0.001
HC → A	0.402	0.096	5.553	0.000
HC → SN	0.262	0.108	3.554	0.000
HC → PBC	0.371	0.083	4.804	0.000
EA → PI	0.153	0.078	2.112	0.035
EA → A	0.346	0.076	5.026	0.000
EA → SN	0.428	0.093	5.639	0.000
EA → PBC	0.333	0.067	4.500	0.000

### 4.3 Mediation effect analysis

To further validate how Health Consciousness (HC) and Environmental Awareness (EA) influence Purchase Intention (PI) through Attitude (A), Subjective Norm (SN), and Perceived Behavioral Control (PBC), this study employed the percentile Bootstrap method with 5,000 resamples for mediation effect analysis. The results, presented in Table 6, show that the indirect effects of all mediation paths reached statistical significance (*p* < 0.01), indicating that Health Consciousness and Environmental Awareness have a significant indirect influence on Purchase Intention through the mediating variables.

**Table 6:** Mediation effect analysis results.

Path	Effect type	Effect value	SE value	*p*-value	95% CI		Conclusion
					Lower	Upper	
HC → A → PI	Indirect effect	0.134	0.025	0.000	0.070	0.170	Partial mediation
HC → SN → PI		0.057	0.019	0.002	0.017	0.090	Partial mediation
HC → PBC → PI		0.084	0.019	0.000	0.038	0.113	Partial mediation
HC → PI	Direct effect	0.315	0.050	0.000	0.217	0.412	
HC → PI	Total effect	0.590	0.051	0.000	0.490	0.690	
EA → A → PI	Indirect effect	0.131	0.025	0.000	0.076	0.173	Partial mediation
EA → SN → PI		0.056	0.021	0.006	0.015	0.094	Partial mediation
EA → PBC → PI		0.084	0.019	0.000	0.043	0.118	Partial mediation
EA → PI	Direct effect	0.236	0.048	0.000	0.142	0.329	
EA → PI	Total effect	0.508	0.050	0.000	0.410	0.606	

Specifically, the total effect of Health Consciousness (HC) on Purchase Intention (PI) is 0.590 (*p* < 0.01), with a direct effect of 0.315 (*p* < 0.01) and an indirect effect of 0.275. This indicates that Health Consciousness not only directly influences Purchase Intention but also exerts an indirect effect through Attitude (A), Subjective Norm (SN), and Perceived Behavioral Control (PBC). Among the indirect paths, the mediation effect of Attitude is the most significant. The mediation effect of HC → A → PI is 0.134 [95% CI: (0.070, 0.170), *p* < 0.01], suggesting that consumers with higher Health Consciousness are more likely to form a positive attitude toward green packaged organic foods, significantly enhancing their purchase intention. The mediation effect of Subjective Norm is 0.057 [95% CI: (0.017, 0.090), *p* < 0.01], indicating that Health Consciousness indirectly promotes purchase intention by increasing consumer sensitivity to social expectations. The mediation effect of Perceived Behavioral Control is 0.084 [95% CI: (0.038, 0.113), *p* < 0.01], showing that Health Consciousness further enhances purchase intention by increasing consumers’ perceived feasibility of the behavior.

The total effect of Environmental Awareness (EA) on Purchase Intention is 0.508 (*p* < 0.01), with a direct effect of 0.236 (*p* < 0.01) and an indirect effect of 0.272. This suggests that Environmental Awareness influences purchase intention both directly and through mediating variables. Among the indirect paths, the mediation effect of Attitude is also significant. The mediation effect of EA → A → PI is 0.131 [95% CI: (0.076, 0.173), *p* < 0.01], indicating that consumers with higher Environmental Awareness are more likely to recognize the environmental benefits of green packaged organic foods, significantly boosting their purchase intention. The mediation effect of Subjective Norm is 0.056 [95% CI: (0.015, 0.094), *p* < 0.01], showing that Environmental Awareness indirectly enhances purchase intention by strengthening consumers’ perception of societal advocacy for green consumption behaviors. The mediation effect of Perceived Behavioral Control is 0.084 [95% CI: (0.043, 0.118), *p* < 0.01], indicating that Environmental Awareness further strengthens purchase intention by boosting consumers’ confidence in the feasibility of the purchasing behavior.

## 5 Conclusion

### 5.1 Key findings and discussion

This study, based on an extended Theory of Planned Behavior (TPB) model, explores the role of health consciousness and environmental awareness in influencing purchase intentions for green packaged organic foods. The findings reveal that both health consciousness and environmental awareness have significant direct effects on consumers’ purchase intentions. Health consciousness enhances consumers’ awareness of the health benefits of organic food, thereby increasing their purchase intentions. Environmental awareness, on the other hand, strengthens consumers’ sense of environmental responsibility, further promoting the development of green consumption behavior. In addition, the study highlights the indirect effect of health consciousness and environmental awareness. Through mediation effect analysis, it was found that these two factors not only have a direct effect on purchase intention but also indirectly influence purchase intentions by impacting psychological variables such as attitude, subjective norms, and perceived behavioral control. Notably, attitude plays a central role in all mediation paths. Both health consciousness and environmental awareness significantly enhance consumers’ purchasing motivation through a positive attitude, thus driving their purchase intentions for green packaged organic foods. This finding emphasizes the dominant role of attitude in green consumption behavior, particularly in how enhancing consumers’ product identification and confidence indirectly influences green consumption decisions. When making purchase decisions, consumers tend to consider both the health benefits and environmental attributes of the product, and these two dimensions play a crucial role in forming a positive purchase attitude.

### 5.2 Research implications

By incorporating health consciousness and environmental awareness, this study extends the application of the TPB model, particularly in the field of green consumption, enhancing the model’s explanatory power regarding consumers’ green purchase intentions. The research demonstrates how health consciousness and environmental awareness, by influencing attitude, subjective norms, and perceived behavioral control, indirectly drive the formation of green consumption behavior. By shedding light on the roles these two variables play in green consumption behavior, the study provides new theoretical insights into understanding the motivations behind green consumption, especially in the purchase decision-making process for green packaged organic foods. For businesses, understanding the mechanisms by which health consciousness and environmental awareness influence consumer behavior can help in developing more effective green marketing strategies and optimizing product design, promotional methods, and market positioning. Companies can enhance consumer perceptions by strengthening the health and environmental attributes of their products, thus promoting positive evaluations of their attitude toward the product. Moreover, through social marketing activities such as celebrity endorsements, green advertising, and social media promotions, businesses can increase consumers’ social identification with green consumption, further driving their intention to purchase green products.

### 5.3 Limitations and future research directions

Despite the valuable findings, this study has several limitations that future research could address. Firstly, the study is primarily based on a sample of consumers from China, and the findings may not fully represent consumer behavior in other regions or cultural contexts. Future studies could expand the sample to include consumers from different countries and cultural backgrounds to test the generalizability of the results. Secondly, the study used cross-sectional data, which does not allow for the examination of the long-term effects of health consciousness and environmental awareness on purchase intentions. Future research could employ a longitudinal design to examine the trends in green purchasing behavior over time, revealing its long-term developmental mechanisms. Additionally, future research could explore other factors that may significantly influence green consumption behavior, such as price sensitivity and brand trust, which could play equally important roles in green consumption decisions. Furthermore, while this study focuses on green packaged organic foods, future research could expand to other areas of green consumer products, such as green home goods, eco-friendly daily use products, and others, to validate the applicability of the research model in different consumption contexts.

## Data Availability

The original contributions presented in the study are included in the article/supplementary material, further inquiries can be directed to the corresponding author.
